# Increased levels of inflammatory markers in the subscapularis tendon and joint capsule in patients with subacromial impingement

**DOI:** 10.1007/s00167-020-05992-9

**Published:** 2020-04-30

**Authors:** Stefanos Farfaras, Leyla Roshani, Jan Mulder, Nicholas Mitsios, Erling K. Hallström, Jüri-Toomas Kartus

**Affiliations:** 1 Department of Research and Development, NU-Hospital Group Trollhättan/Uddevalla, 451 80 Uddevalla, Sweden; 2 Department of Orthopedics, NU-Hospital Group Trollhättan/Uddevalla, Uddevalla, Sweden; 3 Department of Neurosciences, Science for Life Laboratory, Karolinska Institutet, Stockholm, Sweden; 4 Department of Orthopedics, Institute of Clinical Science, Sahlgrenska Academy Gothenburg University, Gothenburg, Sweden

**Keywords:** Subacromial impingement syndrome, Shoulder instability, Shoulder arthroscopy, Cytokines, Pathogenesis, Biopsy analyses

## Abstract

**Purpose:**

To analyze biopsy samples from the subscapularis tendon and from the joint capsule from male patients with subacromial impingement syndrome and compare them with samples from male patients with post-traumatic recurrent shoulder instability, to detect increased inflammatory activity that might be present inside the humeroscapular joint.

**Methods:**

Twenty male patients scheduled for surgery for either subacromial decompression or Bankart reconstruction were included. Four biopsies from each patient were obtained during surgery from the capsule and the subscapularis tendon. Each specimen was analyzed for TNF-α, IL-6, CD-3 and CD-72. Multiplex fluorescence immunohistochemistry was performed on histological samples from the capsule and tendon to demonstrate the level of inflammatory markers. Fluorescence microscope images were acquired using an automated scanning system. On each slide, the number of pixels was registered and used in the analyses.

**Results:**

The subacromial impingement syndrome group comprised eight patients, median age 53 (45–74) years, while the instability group 12, median age 27 (22–48) years (*p* < 0.00001). The amount of IL-6 and TNF-α was significantly higher in the subscapularis tendon of the patients with subacromial impingement syndrome compared with instability patients (*p* = 0.0015 and *p* = 0.0008 respectively). In the capsular samples, significantly higher amount of TNF-α and CD-72 was found in patients with subacromial impingement syndrome compared with instability patients (*p* < 0.0001 for both). On the other hand, the amount of CD-3 was significantly higher in the instability group (*p* = 0.0013).

**Conclusions:**

This study provides evidence that an extended inflammatory process is present, not only in the subacromial bursa but also in the glenohumeral joint in patients with subacromial impingement syndrome.

**Level of evidence:**

Level III.

**Clinical relevance:**

To develop a treatment targeted towards intra-articular inflammatory cytokines appears appealing.

## Introduction

Subacromial impingement syndrome (SAIS) is described as pain provoked in shoulder movements above the horizontal plane [33]. It is one of the most common reasons for shoulder problem consultation [6, 26]. The first description of the syndrome was made in 1972 by Neer, who divided it into three stages. Stage I involves edema and hemorrhage of the bursa and the rotator cuff [34]. This stage can often be difficult to distinguish from the more common secondary impingement syndrome caused by instability. It often appears in persons under 25 years of age. Stage II is characterized by irreversible changes to the cuff, such as fibrosis and tendinitis, and it is often seen in patients between 25 and 40 years of age. In SAIS Stage III, chronic changes, such as partial or complete tears of the rotator cuff, are present. The latter appears in patients of more than 40 years of age. Neer proposed that the syndrome results from direct mechanical conflict between the acromion and the adjacent structures and the rotator cuff [34]. This theory is called the extrinsic theory. Subsequently, another explanation of the syndrome, the intrinsic theory, has been proposed. According to this, the syndrome is a result of chronic degenerative and inflammatory changes in the rotator cuff and the subacromial bursa which lead to the thickening of the subacromial soft tissue, which then results in friction and mechanical conflict in the subacromial space [8, 9, 12, 15, 19, 29, 43, 45].

Several studies have been performed to clarify the pathogenesis of the syndrome. Among other things, this involves studies of the kinematics of the rotator cuff [16–18] and the degeneration of the cuff [8, 10, 12, 15], together with radiographic studies of the configuration of the acromion [3, 31, 39]. There is evidence that an inflammation occurs in the subacromial bursa, due to pro-inflammatory cytokine activation [4, 5, 25, 40, 47]. However, there is little evidence relating to whether or not an inflammatory process is present in only the subacromial space or if it is also present in the adjacent humeroscapular joint.

The purpose of this study was to analyze biopsy samples from the subscapularis tendon and from the joint capsule from male patients with SAIS and compare them with samples from male patients with post-traumatic recurrent shoulder instability, to detect increased inflammatory activity that might be present inside the humeroscapular joint. The future perspective is to use knowledge from the present study to develop new treatment methods for painful shoulders.

The hypothesis of the study was that patients with SAIS would experience an increase in inflammatory mediator expression in their subscapularis tendon and joint capsule compared with patients with post-traumatic recurrent shoulder instability.

## Materials and methods

The patients were referred from the primary care units in the catchment area of NU-Hospital Group, Västra Götaland, Sweden. The study protocol was approved by the Regional Ethics Committee, Västra Götaland, Sweden (IRB: Dnr 076-12).

Patients scheduled for surgery, with either subacromial decompression or Bankart reconstruction, were eligible to participate in the study. To reduce one source of bias, only male patients were asked to participate, due to the inherent difficulty involved in recruiting females with shoulder instability. Furthermore, there is contradictory evidence that the levels of cytokines may vary during the menstruation cycle and/or menopause [13, 27, 28, 32, 41].

The exclusion criteria were female gender, age < 18 years, full-thickness supra- and/or infraspinatus tendon tears and/or macroscopic intra-articular subscapularis tendon tears for the SAIS group, a glenoid fracture larger than a bony Bankart lesion for the patients planned for Bankart reconstruction, co-morbidities such as diabetes, rheumatoid arthritis or osteoarthritis and SAIS Stage III. All the patients gave their written consent, prior to enrollment.

Twenty patients were recruited to the study, based on the power analysis. The enrollment of patients began in April 2012 and ended in June 2013. The study group consisted of eight consecutive patients with SAIS, who were scheduled for arthroscopic subacromial decompression, after having been treated conservatively for at least three months with NSAIDs, subacromial corticosteroid injections and/or physical therapy. The diagnosis was determined with history and clinical tests with a positive painful arc test and positive impingement tests. None of the patients had a full-thickness rotator cuff tear, as determined preoperatively with MRI or ultrasound examination and confirmed macroscopically during arthroscopy. The control group consisted of 12 consecutive patients with post-traumatic recurrent shoulder instability. These patients were the subject of surgical stabilization due to recurrent dislocations. All the subjects had dislocated their shoulder at least three times before referral to an orthopedic specialist. None of the control patients had macroscopic rotator cuff tears.

**Fig. 1:**
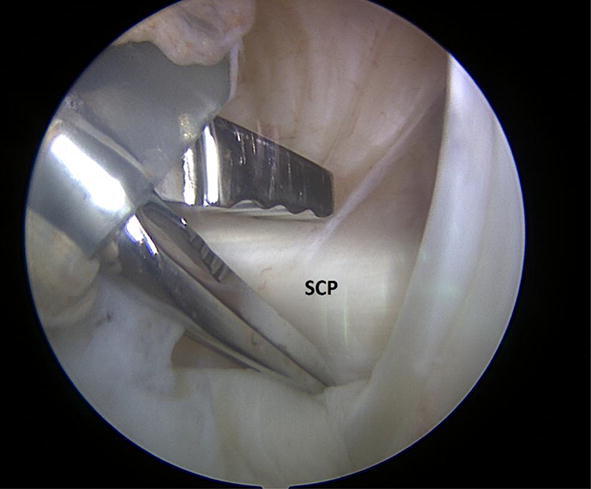
Subscapularis harvesting during the arthroscopic procedure. *SCP subscapularis tendon

**Fig. 2:**
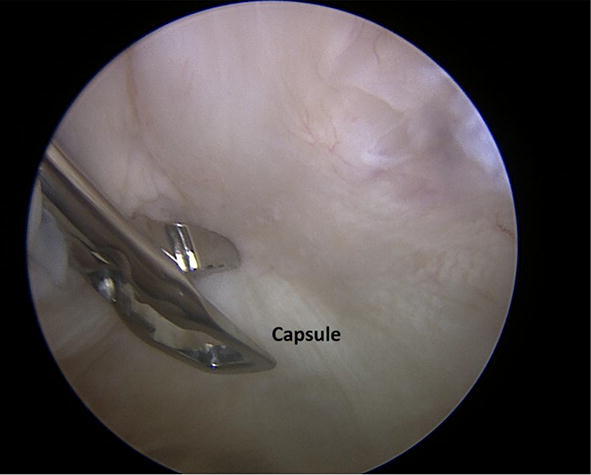
Capsule tissue harvesting during the arthroscopic procedure. *Capsule joint capsule of the ventral part of the shoulder just below the subscapularis tendon

Before the arthroscopic intervention (subacromial decompression for the SAIS group and Bankart reconstruction for the Instability group), a complete diagnostic arthroscopy was performed on each subject. After the diagnostic arthroscopy, four full-thickness biopsies were obtained from the cranial part of the mid-portion of the subscapularis tendon and four from the joint capsule just below the caudal part of the subscapularis tendon (Figs. 1 and 2, respectively). The biopsy samples were harvested with an arthroscopic punch. Their size was approximately 1–2 × 1–2 mm. The specimens were fixed in 4% neutral-buffered formalin immediately after harvest and embedded in paraffin. Each specimen was cut using a sliding microtome and mounted on SuperFrost slides (Histolab Products AB, Sweden). Two identical slides from the most representative sample from each patient were sectioned and mounted.

Each specimen was analyzed for TNF-α, IL-6, CD-3 and CD-72. TNF-α and IL-6 are pro-inflammatory cytokines which are expressed early and play a central role in the inflammatory process [20]. CD-3 and CD-72 are cell surface molecules (Clusters of Differentiation—CD) aiming at T cells and B cells, respectively [2]. T cells are important in the initial stages of inflammation by activating B cells, through the expression of various cytokines, or killing infected cells by transformation into killer T cells (natural killer cells). Once they are activated, the B cells produce various antibodies in the inflammation process.

### Multiplex fluorescence immunohistochemistry

Multiplex fluorescence immunohistochemistry was performed on histological sections from capsule and tendon sections to demonstrate the level of inflammatory markers. All the sections were initially treated on an automated Leica Biosystems (Worldwide) Bond RX system as follows. Briefly, sections were deparaffinized (Bond Dewax solution AR9222, Leica Biosystems, Worldwide), rehydrated and treated for 40 min in an ethylenediaminetetraacetic acid (EDTA)-based pH 9.0 solution (Bond Epitope Retrieval solution 2 AR9640, Leica Biosystems, Worldwide) to unmask the antigens. Slides were subsequently incubated in normal donkey serum for 30 min. Two identical slides from each patient were treated separately using different primary antibodies in two experiments. The slides were incubated overnight at 4 °C. The primary antibodies used in experiment 1 were the T-cell marker CD3E (HPA043955, Atlas Antibodies, Sweden), B-cell marker CD-72 (HPA044658, Atlas Antibodies, Sweden) and IL-6 (HPA060030, Atlas Antibodies, Sweden). In experiment 2, the antibodies were endothelial cell marker CD31 (sc-1506, Santa Cruz Biotechnology, Dallas, USA), TNF-alpha (TNF-α) (HPA064998, Atlas Antibodies, Sweden) and Collagen I (AB6308, Abcam, Worldwide) diluted (1:600, 1:600, 1:800, 1:70, 1:2000 and 1:200, respectively) in Bond Primary antibody diluent (AR9352, Leica Biosystems, Worldwide). After incubation with primary antibodies, the redundant antibodies were washed 3 × 15 min in phosphate-buffered saline (PBS) and incubated for 90 min at room temperature with an appropriate secondary antibody tagged with different fluorophores [anti-mouse, -rabbit or -goat, Fluorescein IsoThioCyanate (FITC)-, Cyanine 3.5 (Cy3.5)- or Cyanine 5 (Cy5)-conjugated] diluted 1:200 in Tris/HCL, NaCl Blocking Buffer (TNB) (Perkin Elmer, Poland). After incubation with secondary antibodies, slides were washed 3 × 15 min in PBS. Furthermore, slides were incubated for 10 min in 1% Sudan Black solution (Sigma/Merck, Germany) in 70% ethanol to quench autofluorescence and mounted in 4′,6-diamidino-2-phenylindole (DAPI)-containing mounting medium (P-36931, Life Technologies, Massachusetts, USA).

### Slide scanning microscopy and image analysis

Fluorescence microscope images were acquired on an automated scanning system (VSlide Scanning Platform, MetaSystems GmbH, Altlussheim, Germany) equipped with a CoolCube 2 camera, 2.5×, 5×, 10× and 20× objectives and filter sets for DAPI (to visualize the cell nuclei in both experiments), FITC (to visualize Collagen I and CD3E in experiments 1 and 2, respectively), Cy3.5 (to visualize CD-3 and CD-72 in experiments 1 and 2, respectively) and Cy5 (to visualize TNF-α and IL-6 in experiments 1 and 2, respectively). Whole microscope slides were scanned at 2.5 × and tissue was detected based on the DAPI signal. After generating a position map, all tissue-covered areas were scanned using a 10 × primary objective (when the signal intensity was not strong enough, 20 × was the primary objective chosen). Individual field of view images were stitched to generate a large four-channel fluorescence image of the entire specimen with microscopic resolution. Fluorescence intensity and distribution of proteins around different parts of tendon structure were analyzed using ImageJ (ImageJ National Institute of Health Software). The various tissue areas were segmented based on the staining patterns of collagen and endothelial cells and information available in the literature [23]. However, the evaluation of the tendon biopsies involved all the parts of the tendon (epitenon, paratenon and endotenon) as a whole. No separate analyses for the different parts of the tendon were made. The size, intensity and distribution of proteins were determined using particle analysis, where the cut-off size and circularity were adjusted accordingly. On each slide, the number of pixels was registered and used in the analyses. The measurement accuracy was one pixel; however, in the results, the mean value of the measurements for each cytokine is reported with an accuracy of one decimal.

CD31 and Collagen I were used for orientation purposes and, as a result, they were not used any further in the analyses.

### Statistics analyses

Mean (SD) or median (range) values are presented when applicable. A *p*-value of < 0.05 was considered statistically significant. All *p*-values are two tailed. For comparisons of the number of pixels and other parametric variables, the unpaired *t*-test was used.

The primary variable in the study was the number of pixels for the different inflammatory markers in the biopsies.

In the power analysis, it was estimated that a difference of one pixel in mean intensity between the groups would be of interest to detect. It was estimated that the standard deviation would be up to eight times the difference between groups. To reach a power of 80%, 1006 counts from each group were required for each comparison.

## Results

The age distribution in the study groups is presented in Table 1.

**Table 1:** Age of the study groups

Diagnosis	Number	Age (years)		
		Mean	Min–max	SD
SAIS	8	57.5	45–74	10.7
Shoulder instability	12	30.4	22–48	8.0
*p*-value	< 0.00001*			

Bold *p*-values indicate a statistically significant difference

*SD* standard deviation

*Patients suffering from SAIS were significantly older than patients with shoulder instability problems

A correlation analysis between age and pixel intensity revealed low and random, both positive and negative, correlations (Table 2).

**Table 2:** The correlation of pixel intensity to the age of the patients

Cytokine	Location	Group	Correlation co-efficient (Rho)
CD-3	Capsule	Pain	− 0.06
CD-3	Capsule	Instab	0.16
CD-3	Tendon	Pain	0.11
CD-3	Tendon	Instab	0.08
CD-72	Capsule	Pain	− 0.07
CD-72	Capsule	Instab	− 0.03
CD-72	Tendon	Pain	− 0.08
CD-72	Tendon	Instab	− 0.18
IL-6	Capsule	Pain	− 0.22
IL-6	Capsule	Instab	− 0.03
IL-6	Tendon	Pain	− 0.12
IL-6	Tendon	Instab	0.04
TNF-alfa	Capsule	Pain	− 0.10
TNF-alfa	Capsule	Instab	− 0.10
TNF-alfa	Tendon	Pain	0.03
TNF-alfa	Tendon	Instab	− 0.06

The amount of IL-6 and TNF-α was significantly higher in the subscapularis tendon of the patients with SAIS compared with instability patients. No significant difference was found regarding CD-3 and CD-72 (Table 3).

**Table 3:** The mean concentration expressed in pixels of CD-3, CD-72, IL-6 and TNF-α in the subscapularis tendon

	CD-3		CD-72		IL-6		TNF-α	
	Mean	SD	Mean	SD	Mean	SD	Mean	SD
SAIS (pixels) Counts	5.7 *n* = 932	6.0	3.3 *n* = 889	2.8	5.8 *n* = 889	13.2	8.5 *n* = 2943	17.4
Shoulder instability (pixels) Counts	5.7 *n* = 2532	16.5	3.1 *n* = 2532	2.6	4.6 *n* = 2532	8.3	7.4 *n* = 5654	12.0
*p*-value	N.s.*		N.s.*		*p* = **0.0015**		*p* = **0.0008**	

Bold *p*-values indicate a statistically significant difference

*n.s non-significant

In the capsular samples, significantly more TNF-α and CD-72 were found in patients with SAIS compared with instability patients, as is shown in Table 4. On the other hand, CD-3 was significantly higher in the instability group. No significant difference in IL-6 was found.

**Table 4:** The mean concentration expressed in pixels of CD-3, CD-72, IL-6 and TNF-α in the capsule

	CD-3		CD-72		IL-6		TNF-α	
	Mean	SD	Mean	SD	Mean	SD	Mean	SD
SAIS (pixels) Counts	5.8 *n* = 3812	11.4	4.2 *n* = 3812	3.5	4.0 *n* = 3812	6.8	7.9 *n* = 7421	12.9
Shoulder instability (pixels) Counts	6.7 *n* = 5767	15.6	3.5 *n* = 5767	3.7	4.3 *n* = 5767	9.3	5.4 *n* = 18,908	8.8
*p*-value	*p* = **0.0013**		*p* < **0.0001**		n.s.*		*p* < **0.0001**	

Bold *p*-values indicate a statistically significant difference

*n.s. non-significant

**Fig. 3:**
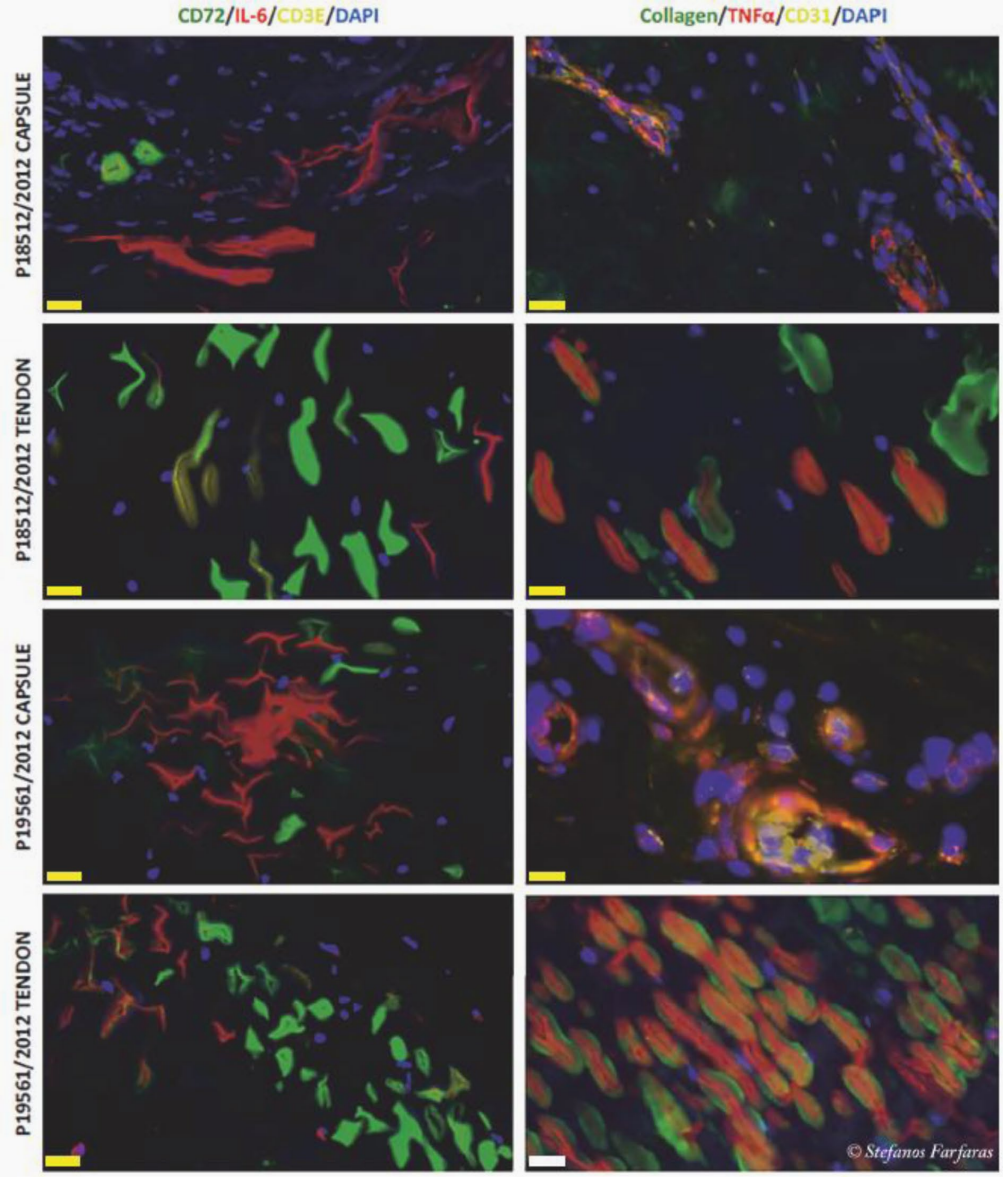
Fluorescence microscope images of the analyzed samples. The figures depict protein expression in capsule and tendon tissue samples from two patients, one from the instability group (P18512/2012) and one from the SAIS group (P19561/2012). Representative staining is shown for the inflammatory molecules TNF-α and IL-6, together with T-cell marker CD3E, B-cell marker CD72, endothelial marker CD31 and Collagen I. CD-31 and Collagen I were used for orientation purposes and, as a result, they were not used any further in the analyses. A general trend towards more robust signaling for TNF-α, IL-6 and CD-72 is evident in cells from the SAIS patients compared with instability patients. Scale bar 5 µm (yellow bar) and 20 µm (white bar)

Figure 3 illustrates the differences in protein expression in the joint capsule and the subscapularis tendon between the instability and the SAIS group patients.

## Discussion

The most important finding in the present study is the presence of increased levels of pro-inflammatory cytokines both in the subscapularis tendon and in the joint capsule in patients with SAIS compared to patients with shoulder instability. This finding suggests that a more widespread inflammatory process is present in patients with SAIS, affecting not just the subacromial bursa but also structures not directly adjacent to it.

The role of pro-inflammatory cytokines has been demonstrated in the subacromial bursa in patients with both rotator cuff disease and other shoulder conditions. Rahme et al. have demonstrated an increase in the number of inflammatory cells (mononuclear cells) in bursal biopsies in patients with SAIS [37]. Increased levels or the expression of TNF-α and IL-6 in the subacromial bursa have been found by Sakai et al. [40], Blaine et al. [4, 5], and Voloshin et al. [47] in patients with rotator cuff tears compared with controls. Shih et al. has also reported increased levels of IL-1β in patients with rotator cuff tears (partial- and full-thickness tears) [42]. Kanbe et al. have shown an increase in the expression of IL-6 around vascular tissue in patients with frozen shoulder and concomitant SAIS (synovial proliferation during arthroscopy at the rotator interval) [22]. It has also been reported by other authors that frozen shoulder may develop secondary to subacromial bursitis [35]. Furthermore, increased levels of IL-8 have been reported by Okamura et al. [36] and dysregulation of cytokines expression has been described by Akbar et al., in patients with frozen shoulder [1]. In the present study, increased levels of pro-inflammatory cytokines were found in the shoulder joint capsule and subscapularis tendon. This implies that the source of pain may be a diffuse inflammatory process which extends beyond the subacromial bursa. As the patients included in the present study had no perforating rotator cuff tears, it is unlikely that the increased expression of cytokines in the specimens was due to proliferation/affection from the inflamed subacromial bursa. This finding must, therefore, be regarded as a local inflammation. Gotoh et al. [14] have demonstrated increased levels of IL-1β in the glenohumeral synovia in patients with rotator cuff disease and perforating rotator cuff tears compared with those with non-perforating tears. This finding is in line with the results in the present study, even though the increase in Gotoh’s study may be due to the proliferation of cytokines through the perforating tear. Furthermore, in Gotoh’s study, no control group with a healthy tendon was analyzed.

To date, the exact role of various pro-inflammatory cytokines in the human body has not been investigated in detail. It is known that IL-1β is expressed in the early stages of inflammation together with TNF-α and induces the production of IL-6 [20]. Furthermore, Tsuzaki et al. have demonstrated that IL-1β induces IL-6, among other factors, in the flexor digitorum profundus specimens in normal controls [44]. These data support the findings in the present study that TNF-α and IL-6 play a role in the inflammation of the subscapularis tendon and joint capsule in SAIS patients. Increased levels of IL-8 and IL-18 have been found in the synovial fluid of patients with OA of the shoulder joint compared to healthy individuals [46]. It is possible that, in a similar pattern, more cytokines, such as IL-8 or IL-18, may also be increased in SAIS patients. It was, however, decided in the present study to determine the cytokines to be analyzed in advance and limit the number of analyses to TNF-α as a pro-inflammatory cytokine and to IL-6 which is expressed later in the inflammation cascade process [20] and not to screen for whichever cytokines might be present.

Another finding in the present study is the increased amount of CD-72 in the capsule of SAIS patients. CD-72 is a glycoprotein involved in B-cell proliferation and differentiation [38]. Furthermore, it is a B-cell receptor [21]. This indicates an increase in the activity and proliferation of B-cells in the synovial tissue of the joint. The increased level of pro-inflammatory cytokines and B-cell activators in the subscapularis tendon and joint capsule of SAIS patients in the present study suggests a more comprehensive inflammation than in the subacromial bursa alone. This may be due to the endocrine action of the cytokines in question or to diffusion into the adjacent tissues. It can therefore be assumed that the inflammation that occurs in the subacromial bursa has a distal effect even towards the joint capsule.

Subacromial corticosteroid injections and NSAIDs have been broadly used in the management of subacromial impingement. As it appears that the inflammation is not only localized to the subacromial bursa alone, but also is spread to adjacent structures. Treating these structures as well might also be indicated. A selective and effective factor, such as a cytokine antagonist towards TNF-α and IL-6, may be of interest. This would offer a target-specific therapy and would possibly avoid the side effects of corticosteroid use. To the author’s knowledge, no treatment of this kind has so far been applied.

This study has several limitations. Firstly, the number of cytokines and the role of each of them in the inflammation cascade are not well known. Only some, albeit predetermined, cytokines involved in this process were analyzed. This may represent a risk that an analysis of other cytokines might have led to another conclusion. It is possible that other cytokines, such as IL1, IL-8 or IL-18, are also increased in SAIS patients. Furthermore, there is an inherent difference in the age of the study groups. This was, unfortunately, inevitable, due to the prevalence of shoulder instability in younger individuals. A control group of age-matched healthy individuals would naturally have been better, but this was impossible for ethical reasons. The age difference may have altered the results towards an increase in cytokines in older individuals. There is limited evidence of this and most results refer to blood measurements and not to local tissue samples, as in the present study. Ferucci et al. have found progressively increasing levels of IL-6 (among other cytokines) with age but no increase in TNF-α in blood sample analyses [11]. These findings are, however, difficult to interpret, as it is not known whether this increase in cytokine levels with age is inherent or secondary to co-morbidities (such as cardiovascular disease, diabetes, arthritis, Alzheimer disease) [7, 24, 30]. Taken together, there is diverging evidence relating to what happens to cytokines during aging. The correlation analysis in the present study revealed only low and random, both positive and negative, correlations between age and pixel intensity. This indicates that age did not act as a bias factor in the present study. Additionally, the results of the present study only refer to male patients, which, due to cyclical hormonal changes in females, might not be a true weakness. As always, it is necessary to consider that the study maybe was under-powered, in spite of the large number of pixel counts registered.

The strengths of this study include the fact that cytokines were measured in tissue specimens from the affected area and not in the bloodstream and that it was performed on humans.

## Conclusion

This study provides evidence that an extended inflammatory process is present, not only in the subacromial bursa but also in the glenohumeral joint in patients with SAIS. Therefore, a treatment targeted towards intra-articular inflammatory cytokines appears appealing.

## Abbreviation

Cy3.5: Cyanine 3.5
Cy5: Cyanine 5
EDTA: Ethylenediaminetetraacetic acid
FITC: Fluorescein Isothiocyanate
SAIS: Subacromial impingement syndrome
TNB: NaCl blocking buffer

## References

[1] Akbar M, McLean M, Garcia-Melchor E, Crowe LA, McMillan P, Fazzi UG (2019). Fibroblast activation and inflammation in frozen shoulder. PLoS ONE.

[2] Bernard A, Boumsell L (1984). The clusters of differentiation (CD) defined by the first international workshop on human leucocyte differentiation antigens. Hum Immunol.

[3] Bigliani LU (1986). The morphology of the acromion and its relationship to rotator cuff tears. Orthop Trans.

[4] Blaine TA, Cote MA, Proto A, Mulcahey M, Lee FY, Bigliani LU (2011). Interleukin-1beta stimulates stromal-derived factor-1alpha expression in human subacromial bursa. J Orthop Res.

[5] Blaine TA, Kim YS, Voloshin I, Chen D, Murakami K, Chang SS (2005). The molecular pathophysiology of subacromial bursitis in rotator cuff disease. J Shoulder Elbow Surg.

[6] Bot SD, van der Waal JM, Terwee CB, van der Windt DA, Schellevis FG, Bouter LM (2005). Incidence and prevalence of complaints of the neck and upper extremity in general practice. Ann Rheum Dis.

[7] Bruunsgaard H, Pedersen M, Pedersen BK (2001). Aging and proinflammatory cytokines. Curr Opin Hematol.

[8] Castagna A, Cesari E, Gigante A, Conti M, Garofalo R (2013). Metalloproteases and their inhibitors are altered in both torn and intact rotator cuff tendons. Musculoskelet Surg.

[9] Factor D, Dale B (2014). Current concepts of rotator cuff tendinopathy. Int J Sports Phys Ther.

[10] Farfaras S, Ejerhed LE, Hallstrom EK, Hultenby K, Meknas K, Movin T (2018). More histologic and ultrastructural degenerative signs in the subscapularis tendon and the joint capsule in male patients with shoulder impingement. Knee Surg Sports Traumatol Arthrosc.

[11] Ferrucci L, Corsi A, Lauretani F, Bandinelli S, Bartali B, Taub DD (2005). The origins of age-related proinflammatory state. Blood.

[12] Garofalo R, Cesari E, Vinci E, Castagna A (2011). Role of metalloproteinases in rotator cuff tear. Sports Med Arthrosc.

[13] Gazvani R, Templeton A (2002). Peritoneal environment, cytokines and angiogenesis in the pathophysiology of endometriosis. Reproduction.

[14] Gotoh M, Hamada K, Yamakawa H, Yanagisawa K, Nakamura M, Yamazaki H (2002). Interleukin-1-induced glenohumeral synovitis and shoulder pain in rotator cuff diseases. J Orthop Res.

[15] Goutallier D, Postel JM, Van Driessche S, Voisin MC (2005). Histological lesions of supraspinatus tendons in full thickness tears of the rotator cuff. Rev Chir Orthop Reparatrice Appar Mot.

[16] Hallstrom E, Karrholm J (2008). Kinematic evaluation of the Hawkins and Neer sign. J Shoulder Elbow Surg.

[17] Hallstrom E, Karrholm J (2006). Shoulder kinematics in 25 patients with impingement and 12 controls. Clin Orthop Relat Res.

[18] Hallstrom E, Karrholm J (2009). Shoulder rhythm in patients with impingement and in controls: dynamic RSA during active and passive abduction. Acta Orthop.

[19] Hashimoto T, Nobuhara K, Hamada T (2003). Pathologic evidence of degeneration as a primary cause of rotator cuff tear. Clin Orthop Relat Res.

[20] Hopkins SJ (2003). The pathophysiological role of cytokines. Leg Med (Tokyo).

[21] Ishida I, Kumanogoh A, Suzuki K, Akahani S, Noda K, Kikutani H (2003). Involvement of CD100, a lymphocyte semaphorin, in the activation of the human immune system via CD72: implications for the regulation of immune and inflammatory responses. Int Immunol.

[22] Kanbe K, Inoue K, Inoue Y, Chen Q (2009). Inducement of mitogen-activated protein kinases in frozen shoulders. J Orthop Sci.

[23] Kannus P (2000). Structure of the tendon connective tissue. Scand J Med Sci Sports.

[24] Kiecolt-Glaser JK, Preacher KJ, MacCallum RC, Atkinson C, Malarkey WB, Glaser R (2003). Chronic stress and age-related increases in the proinflammatory cytokine IL-6. Proc Natl Acad Sci USA.

[25] Kim YS, Bigliani LU, Fujisawa M, Murakami K, Chang SS, Lee HJ (2006). Stromal cell-derived factor 1 (SDF-1, CXCL12) is increased in subacromial bursitis and downregulated by steroid and nonsteroidal anti-inflammatory agents. J Orthop Res.

[26] Linsell L, Dawson J, Zondervan K, Rose P, Randall T, Fitzpatrick R (2006). Prevalence and incidence of adults consulting for shoulder conditions in UK primary care; patterns of diagnosis and referral. Rheumatology (Oxford).

[27] Makinoda S, Mikuni M, Sogame M, Kobamatsu Y, Furuta I, Yamada H (1996). Erythropoietin, granulocyte-colony stimulating factor, interleukin-1 beta and interleukin-6 during the normal menstrual cycle. Int J Gynaecol Obstet.

[28] Mangioni S, Vigano P, Florio P, Borghi O, Vignali M, Petraglia F (2005). Effect of activin A on tumor necrosis factor-alpha/intercellular adhesion molecule-1 pathway in endometrial stromal cells. Eur J Obstet Gynecol Reprod Biol.

[29] McFarland EG, Maffulli N, Del Buono A, Murrell GA, Garzon-Muvdi J, Petersen SA (2013). Impingement is not impingement: the case for calling it "Rotator Cuff Disease". Muscles Ligaments Tendons J.

[30] Michaud M, Balardy L, Moulis G, Gaudin C, Peyrot C, Vellas B (2013). Proinflammatory cytokines, aging, and age-related diseases. J Am Med Dir Assoc.

[31] Natsis K, Tsikaras P, Totlis T, Gigis I, Skandalakis P, Appell HJ (2007). Correlation between the four types of acromion and the existence of enthesophytes: a study on 423 dried scapulas and review of the literature. Clin Anat.

[32] Naz RK, Thurston D, Santoro N (1995). Circulating tumor necrosis factor (TNF)-alpha in normally cycling women and patients with premature ovarian failure and polycystic ovaries. Am J Reprod Immunol.

[33] Neer CS (1972). Anterior acromioplasty for the chronic impingement syndrome in the shoulder: a preliminary report. J Bone Joint Surg Am.

[34] Neer CS (1983). Impingement lesions. Clin Orthop Relat Res.

[35] Nobuhara K, Sugiyama D, Ikeda H, Makiura M (1990). Contracture of the shoulder. Clin Orthop Relat Res.

[36] Okamura K, Kobayashi T, Yamamoto A, Shitara H, Osawa T, Ichinose T (2017). Shoulder pain and intra-articular interleukin-8 levels in patients with rotator cuff tears. Int J Rheum Dis.

[37] Rahme H, Nordgren H, Hamberg H, Westerberg CE (1993). The subacromial bursa and the impingement syndrome. A clinical and histological study of 30 cases. Acta Orthop Scand.

[38] Robinson WH, Landolfi MM, Parnes JR (1997). Allele-specific expression of the mouse B-cell surface protein CD72 on T cells. Immunogenetics.

[39] Roidis NT, Motamed S, Vaishnav S, Ebramzadeh E, Karachalios TS, Itamura JM (2009). The influence of the acromioclavicular joint degeneration on supraspinatus outlet impingement and the acromion shape. J Orthop Surg (Hong Kong).

[40] Sakai H, Fujita K, Sakai Y, Mizuno K (2001). Immunolocalization of cytokines and growth factors in subacromial bursa of rotator cuff tear patients. Kobe J Med Sci.

[41] Scholl B, Bersinger NA, Kuhn A, Mueller MD (2009). Correlation between symptoms of pain and peritoneal fluid inflammatory cytokine concentrations in endometriosis. Gynecol Endocrinol.

[42] Shih CA, Wu KC, Shao CJ, Chern TC, Su WR, Wu PT (2018). Synovial fluid biomarkers: association with chronic rotator cuff tear severity and pain. J Shoulder Elbow Surg.

[43] Takase K, Yamamoto K (2005). Histological and ultrastructural changes in the undersurface of the acromion with subacromial impingement. Acta Orthop.

[44] Tsuzaki M, Guyton G, Garrett W, Archambault JM, Herzog W, Almekinders L (2003). IL-1 beta induces COX2, MMP-1, -3 and -13, ADAMTS-4, IL-1 beta and IL-6 in human tendon cells. J Orthop Res.

[45] Tuoheti Y, Itoi E, Pradhan RL, Wakabayashi I, Takahashi S, Minagawa H (2005). Apoptosis in the supraspinatus tendon with stage II subacromial impingement. J Shoulder Elbow Surg.

[46] Wanner J, Subbaiah R, Skomorovska-Prokvolit Y, Shishani Y, Boilard E, Mohan S (2013). Proteomic profiling and functional characterization of early and late shoulder osteoarthritis. Arthritis Res Ther.

[47] Voloshin I, Gelinas J, Maloney MD, O'Keefe RJ, Bigliani LU, Blaine TA (2005). Proinflammatory cytokines and metalloproteases are expressed in the subacromial bursa in patients with rotator cuff disease. Arthroscopy.

